# Regulation of alveolar macrophage death in pulmonary fibrosis: a review

**DOI:** 10.1007/s10495-023-01888-4

**Published:** 2023-09-14

**Authors:** Ganghao Yang, Yang Yang, Yiping Liu, Xiaoshu Liu

**Affiliations:** 1 Department of Respiratory and Critical Medicine, University of Electronic Science and Technology of China Sichuan Provincial People’s Hospital, Sichuan Academy of Medical Sciences and Sichuan People’s Hospital, Chengdu, Sichuan China; 2 Department of Respiratory and Critical Medicine, Peking Union Medical College Hospital, Chinese Academy of Medical Sciences and Peking Union Medical College, No. 1 Shuai Fu Yuan Street, Dong Cheng District, Beijing, 100730 China

**Keywords:** Pulmonary fibrosis, Macrophage polarization, Classically activated (M1) macrophages phenotypes, Alternatively activated (M2) macrophages phenotypes, Pyroptosis

## Abstract

Pulmonary fibrosis (PF) is a disease in which excessive extracellular matrix (ECM) accumulation occurs in pulmonary mesenchyme, which induces the destruction of alveolar structures and poor prognosis. Macrophage death is responsible for ECM accumulation after alveolar epithelial injury in PF. Depending on the local micro-environments, macrophages can be polarized to either classically activated (M1) or alternatively activated (M2) macrophage phenotypes. In general, M1 macrophages can promote inflammation and sterilization, stop the continuous damage process and prevent excessive repair, while M2 macrophages are anti-inflammatory and promote tissue repair, and excessive M2 macrophage activity may inhibit the absorption and degradation of ECM. Emerging evidence has revealed that death forms such as pyroptosis mediated by inflammasome affect polarization direction and ultimately lead to the development of PF. Pharmacological manipulation of macrophages death signals may serve as a logical therapeutic strategy for PF. This review will focus on the current state of knowledge regarding the regulation and underlying mechanisms of macrophages and their mediators in the influence of macrophage death on the development of PF. We expect to provide help in developing effective therapeutic strategies in clinical settings.

## Background

Pulmonary fibrosis (PF) is a progressive disease that can raise the mortality and the rate of disability of patients with lung disease. The clear mechanism of the pathological procedure is still unknown, while there lots of advances in past decades. Macrophage, with the ability to polarize into different phenotypes, is an innate immunological cell that plays a contradictory but connected role in pulmonary fibrosis. How the dead form of cells participates in fibrosis by effect macrophages has attracted researchers a lot whereas the association between macrophages and cell death is in a mess yet. This review aims to figure out the underlying interrelationship between macrophage and cell death form and expounds a possible mechanism of pulmonary fibrosis from a novel sight.

## Introduction

Pulmonary fibrosis (PF) is the most common fibrosing lung disease with poor prognosis and no effective treatment [1]. It is characterized by the development of excessive ECM deposition, leading to decreased static lung compliance, disrupted gas exchange and respiratory failure and death [2]. The median survival time for patients with idiopathic pulmonary fibrosis (IPF), the most classical disease of PF, is approximately 3 years after initial diagnosis due to unsatisfactory effect of current anti-fibrosis drugs [3].

Previous studies have confirmed that excessive deposition of ECM is an important factor in the progression of PF [4]. The formation of ECM is a pathological process of abnormal repair after alveolar epithelial cells (AECs) injury [5]. Therefore, it is very important to control the inflammatory response and carry out normal repair of the damaged tissue. In normal tissue repair, macrophages are important cells to degrade and absorb ECM [6]. Macrophages are mainly involved in tissue damage repair in the immune regulatory pathway of the human body [7]. Bone marrow monocytes migrate into tissues and become tissue macrophages [8]. At this time, the macrophages are in a dormant state, which is called resting-state (M0) macrophages [9]. When AECs are injured, monocytes gather in the lung interstitium [10]. Under the influence of pro-inflammatory factors, M0 macrophages differentiate into classically activated (M1) macrophages [7]. Once activated, M1 macrophages will produce tumor necrosis factor-α (TNF-α), L-1β and oxygen free radicals to fight infection or remove foreign substances, thus terminating the damage repair process and preventing excessive repair [9]. However, the differentiation of M0 into alternatively activated(M2) macrophages can be over-anti-inflammatory and promote abnormal tissue repair [11]. This excessive activity of M2 macrophages may lead to the occurrence of various fibrosis diseases [9]. In conclusion, macrophages play an important role in maintaining the homeostasis of lung tissue, and the maladjustment of the polarization direction of macrophages can lead to the occurrence of PF.

The direction of macrophage polarization is controlled by different cell death forms such as apoptosis, pyroptosis and autophagy. These death forms are also closely related to PF [12–14]. Pyroptosis is a process of programmed cell death mediated by classical inflammasome pathway [15]. The activation of inflammasome pathway will induce macrophages to gather and produce cascade inflammatory reaction [16]. The lysed macrophages will not only promote the secretion of cytokines such as IL-1 and IL-18, but also lead to TGF-β1 secreted by M2 macrophages, which promotes the ECM deposition and the inflammatory process in PF [9]. Autophagy can also regulate the polarization direction of macrophages and participate in the regulation of inflammatory response [12]. Autophagy is an evolutionarily conserved and genetically regulated pathway that serves to degrade and clear subcellular components [12, 17]. Excessive macrophage autophagy will increase the occurrence of apoptosis, promote the transformation from M1 to M2, and degrade the activation of NF-κB (nuclear factor-kappa B) inflammatory signal pathway, inhibiting the production and release of proinflammatory factor TNF-α, IL-6, IL-1β and IL-12 [18]. Finally, the above process affected the degradation of ECM and promoted the abnormal repair [12]. Therefore, autophagy is involved in PF by regulating the direction of polarization of macrophages and regulating cytokine secretion.

Emerging evidence has revealed that macrophages death plays important roles in influencing the progression of PF [10]. There is increasing recognition that inflammation and cell death reciprocally affect each other and form an auto-amplification loop of these two factors, which in turn exaggerates fibrosis [19]. Therefore, pharmacological manipulation of macrophages death signals may potentially serve as a logical therapeutic strategy for PF. This review will focus on recent advances in the regulation of AM death and underlying mechanisms on the development of PF.

## Analysis of macrophages in human PF lungs

The distinction of the composition of macrophages in human fibrosis pulmonary lungs, compared with normal people, has been reported [20–22]. Researchers analyzed the composition of macrophages in fibrosis lung, finding that resident alveolar macrophages and monocytes-derived macrophages, which however now can be further classified into 3 or 4 populations via single-cell RNA-sequencing(scRNA-seq), comprise the major macrophage population [21]. In 2018, researchers first described that there were at least 2 distinct macrophage populations associated with the process of PF, which is both highly expressing genes SPP1 and CHI3L1 [21]. Furthermore, a study suggested that only the SPPS1himacrophages which notably express MERTK were increased in the lung tissues which indicated this subpopulation of macrophages would be the potential highlight of a therapeutic field in PF [23].

Scholars applied a self-organizing map algorithm (FlowSOM) to identify which subsets of myeloid cells participate in fibrosis, revealing that the myeloid lineage cells in IPF can be divided into functional six subsets: monocyte-like cells, monocyte-derived macrophages, monocyte-derived dendritic cells, interstitial macrophages, dendritic cells and alveolar macrophages [24]. Amid them, monocyte-like cells, monocyte-derived macrophages (CD206-subsets) and interstitial macrophages (CD206-subsets) are decreased in IPF lungs. In addition, researchers observed that the AMs from patients with IPF over-expressed a range of regulative genes like ITGB1, ITGB2, S100A8, and FN1 [24]. Collectively, by analysis, the diversity of macrophages in IPF, and specific pathological mechanisms of PF would be deeply clarified in the future.

### Role of macrophages in PF

#### Macrophage-derived secretory proteins and PF

In any phase of PF, macrophages mediate different immunocytes to participate in the process of fibrosis via releasing a variety of proteins, like growth factors, chemokines, and enzymes [25]. TGF-β, some of which is released by macrophages, as the most major effective profibrotic factor is widely researched [26]. It is positive feedback that cells exposed to TGF-β secrete more of this cytokine, interestingly [25]. Besides, macrophages secrete platelet derived growth factor (PDGF), vascular endothelial growth factor (VEGF) and insulin like growth factor-1 (IGF-1) to advance the proliferation of fibroblasts and the synthesis of collagen [27]. Nintedanib, an intracellular inhibitor of tyrosine kinase receptor, has been demonstrated to hinder fibrosis by targeting PDGF, VEGF and FGF (fibroblast growth factor) and is considered as a novel pharmacological treatment [28, 29]. Macrophages can release chemokine (C-C motif) ligand-2 (CCL2) and chemokine (C-C motif) ligand-18 (CCL18) to recruit circulation-derived monocyte to aggravate fibrosis, which is similar to IL-1β [30]. Nevertheless, macrophages, with the capability of secreting MMPs (matrix metalloproteinases) to promote the degradation of ECM, can abate fibrosis [31, 32]. Quite a few researches prove that the expression of MMP-3 augments in the lung tissue of patients with PF, which induces fibroblast activation and epithelial interstitial transformation [31, 33, 34]. In contrast, MMP-3 can not only activate TGF-β to exacerbate fibrosis but show the ability to encumber IGF-1 to execute the role of anti-fibrosis [31]. Macrophages rely on those secretory proteins, showing distinct functions, some of which even are contradictory [7]. This phenomenon may relate to different micro-environment to which macrophages exposure and to the different subpopulations of macrophages which would be further elucidated following.

#### Macrophage polarization in PF

**Fig. 1:**
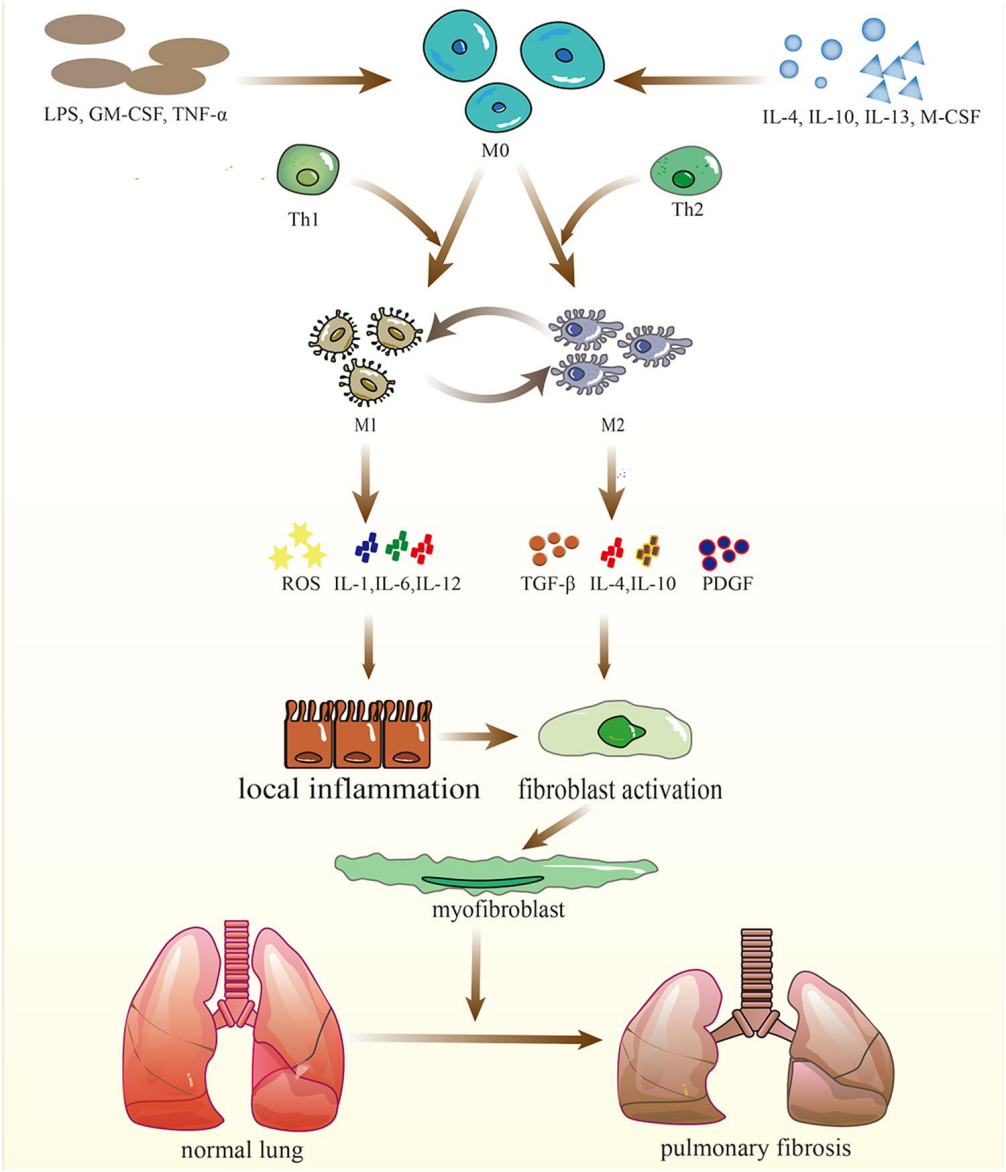
M0 can polarity into M1 and M2 by distinct stimuli. M1 plays an inflammatory role by releasing ROS, IL-1, IL-6, and IL-12 while M2 has the profibrotic potential with the releasing of TGF-β, IL-4, IL-10 and PDGF in PF

Macrophage activation is periodical which indicates the plastic feature of macrophages [35]. In the process of macrophage activation, macrophages can differentiate into two functionally distinct sub-phenotypes which differ in inducing factors and expression of surface markers (Fig. 1). Firstly, the M1 macrophage is involved in PF mainly in the early stages of injury to mediate the inflammatory response, basically induced by lipopolysaccharide (LPS), granulocyte-macrophage colony stimulating factor (GM-CSF), IFN-γ, TNF-α [36]. M2 macrophages can release several profibrotic cytokines and newly study points out that M2 can show phenotype of M1 by giving LPS and IFN-γ in vitro culture, additionally finding that the fibrosis of model mice was alleviated [35, 37]. That may be a potential way of treating PF. However, researchers tended to consider both M1 and M2 in the pathogenesis of PF in recent years [10]. Functionally, M1 macrophages, which show an excellent ability to mediate tissue injury, contribute to boosting the immunoreaction of the host by releasing intracellular and pro-inflammatory cytokines and chemokines, like TNF-α, IL-1, IL-6 and IL-12 and the removal of pathogens through generating the ROS (active oxygen species) [38]. At the early stage of inflammation, M0 can transform into M1 induced by inducers like LPS, IFN-γ and GM-CSF [9, 39]. Normally, M1 macrophages promote an inflammatory response in lung air space and then re-transform into M2 macrophages which play an important role in wound healing or anti-inflammation [7]. However, without appropriate termination, M1 will cause excessive inflammation and exacerbate the injury of the AECs via the cytotoxic and pro-inflammatory effects, finally causing aberrant fibroblast proliferation and hypernomic ECM deposition [9]. M2 macrophages are used to be considered the main effect cell in the repair or fibrosis of tissue injury and can release pro-fibrotic cytokines such as TGF-β, IL-4, IL-10 and PDGF, which promote transformation and proliferation of fibroblast and myofibroblast [40]. Researchers used to establish an early PF model via bleomycin, finding that the depletion of M2 will abate PF [41]. Collectively, the unbalance of the proportion of M1 with M2 play an important role in the development of PF.

### Pyroptosis in macrophage drived PF

#### Pyroptosis is a form of regulated cell death

Pyroptosis is defined as inflammatory programmed cell necrosis which induces DNA damage and chromatin condensation, having the ability to protect the host from the infection of pathogenic bacteria or other non-infection damage by inducing local inflammation [15]. However, because of the fleetly numerous pro-inflammatory cytokines released, and the inflammasome dependency, pyroptosis is mechanistically distinct from apoptosis and any other forms of cell death [42]. When cells are stimulated by inflammatory or injury factors, intracellular Caspase can be activated by the corresponding pathway and cleavage pro-gasdermin, pro-IL-1β and pro-IL-18, ultimately lead to cytomemobrane rupture and release of IL-1β and IL-18 [15, 43]. The assembly of inflammasome plays an extraordinarily important role in this progression [44]. Inflammasomes are a kind of cytosolic multi-protein platform and are constituted by at least three main components, including nod-like receptors (NLRs), pro-caspase-1, and apoptosis-associated spot-like proteins (ACS) [16]. The NLRs family, which is prominent basis of the classification of the inflammasome, is a cytoplasmic group of  pattern recognition receptors (PRRs) and characteristic proteins contained at the N-termini of NLRs subdivide these receptors into at least four subfamilies: NLRAs (with a trans-activating domain at the end), NAIPS (with apoptosis inhibitory repeat at the N terminal), NLRPs (with pyrin binding domain at the N terminal) and NLRCs (with Caspase recruitment domain protein, CARD, at the end) [45].

Depending on the distinction between the activated enzyme and the main procedure, pyroptosis can be divided into two pathways [15]. Firstly, the canonical inflammasome pathway characteristically requires the activation of the enzyme of caspase-1 [42, 43, 46, 47]. However, the activation of capcase-1 depends on the assembly of inflammasome which is activated by different upstream pathways [45]. Different types of stimuli, including double-stranded DNA, and bacterial LPS act on NLRs (like the NOD-like receptor thermal protein domain associated protein 3, NLRP3) and spark downstream procedure, those phosphorylated NLRP3 recruit CARD-containing ACSs, which further recruit pro-caspase-1 through their CARDs and the three of them assemble to form the inflammasome, which converts the pro-caspase-1 into caspase-1 [43]. Converted caspase-1 can simultaneously cleavage pro-IL1β, IL-18 and gasdermin D (GSDMD), producing bioactive IL1β, IL-18 and two snippets of GSDMD (N-terminal and T-terminal GSDMD) [47]. And the NT-GSDMD oligomerizes and forms plasma-membrane pores which leads to the fatal release of proinflammatory intracellular contents in the end [15, 42, 47]. Differing from the canonical pathway of pyroptosis, the upstream PRRs are unnecessary for the non-canonical pathway [15, 48]. Recent studies further indicated that both caspase-4 and caspase-5 in humans can be directly triggered via binding to cytosolic LPS [49, 50]. Caspase-11/4/5 cannot directly mature pro-IL-1β and IL-18, unexpectedly, they can mediate the secretion of IL-1β/ IL-18 via NLRP3/caspase-1 pathway and can cleave GSDMD, resulting in pyroptosis eventually [51, 52].

#### Pyroptosis-derived cytokines in macrophage

Pyroptosis happening in macrophages can cause the release of intracellular cytokines which have an extraordinary ability on leading to inflammation [43]. Those cytokines including IL-1β and IL-18 both exist in macrophages in precursor form and will be cleaved once the caspase is mature [53].

IL-1β, which shows robust relevance to inflammation, participates in a variety of immune phenomena like the migration of monocyte, fever of a host and expression of various chemokines [53, 54]. As to IL-18, it plays an important role in the expression of IFN-γ and activation of Th cells and other immune cells [55]. Dimeric caspase on the inflammasome can cleave the pro-IL-1β, pro-IL-18 and gasdermin meanwhile, and the N-terminal from the cleaved gasdermin form a pore on the cell membrane which leads to k + efflux and cell swelling and result in the releasing of IL-1β and IL-18 [47]. New research has suggested that LPS can bind with and directly activate caspase-4/5/11 without the need for PPRs, while caspase-4/5/11 cannot cleave the pro-IL-1β and pro-IL-18 but via the NLRP3 pathway to activate the caspase-1 to mature those cytokines [47, 56]. Additionally, researchers reported that LPS activates caspase-1-dependent pyroptosis by binding to toll-like receptor-4 (TLR4) expression on the macrophage surface, subsequently upregulating IL-1RI expression on macrophages through NF- kB signaling pathway, increasing macrophage sensitivity to IL-1β and promoting assemble of the inflammasome, thus further promoting pyroptosis [57]. Differing from those pathways mentioned above, high mobility group box 1 (HMGB1), which is released from cells in the injury area, binds to the receptor for advanced glycation end products the receptor for advanced glycation end products (RAGE), inducing the endocytosis of HMGB1 and formation of ACS which activates the caspase-1 later [58–60]. Significantly, some studies showed that the assembly of inflammasome and activation of caspase not consequentially induce pyroptosis but cause necroptosis and the mechanism of this phenomenon is still in the mist [61, 62].

#### Macrophage pyroptosis in PF

**Fig. 2:**
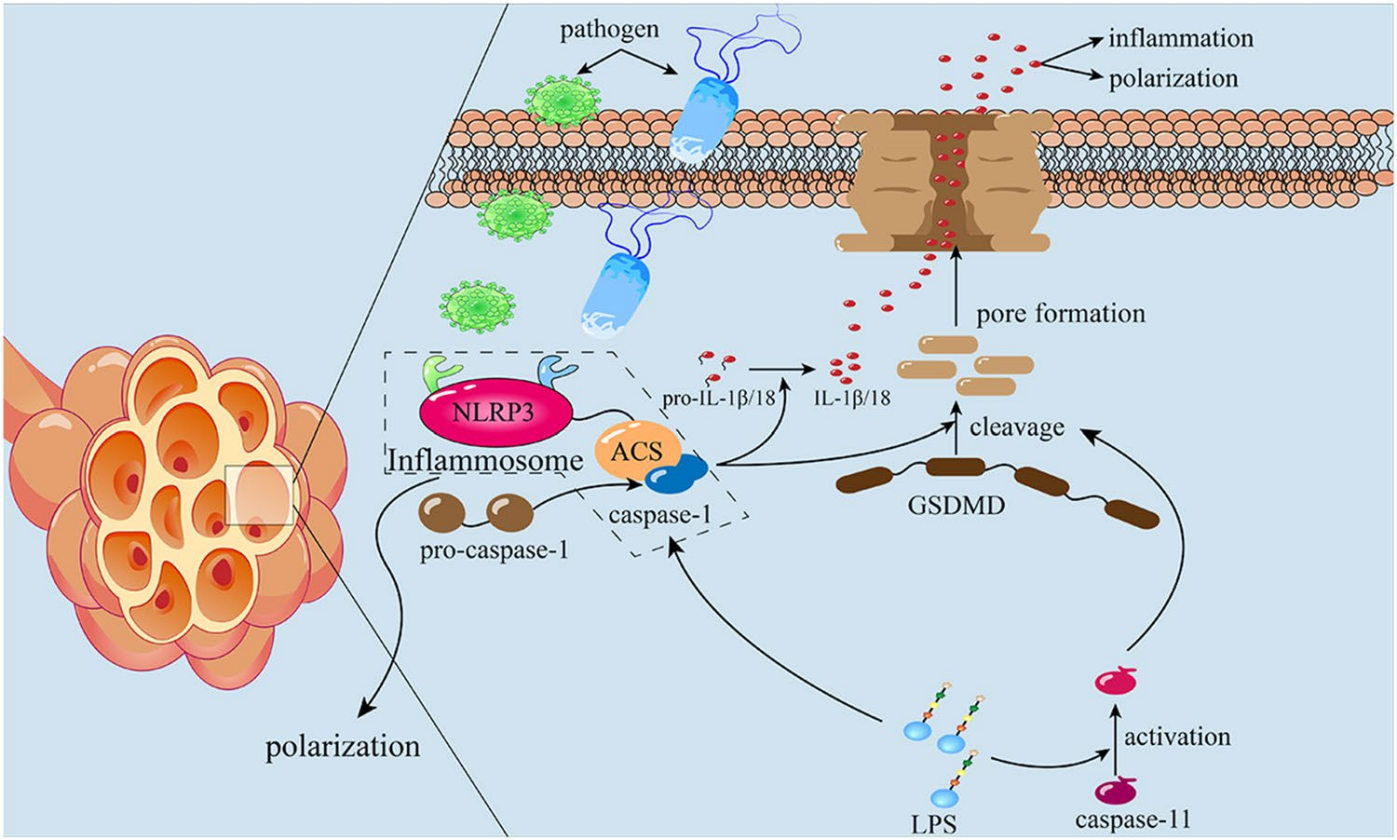
Pyroptosis has two main pathways: canonical pathway and non-canonical pathway. The canonical pathway is regulated by inflammasome assembly which is mainly activated by PAMPs and DAMPs. Active NLRP3 binds pro-caspase-1 via ACS to form the NLRP3-inflammasome, further hydrolyzing and activating pro-caspase-1. Caspase-1 of dimerization cleaves GSDMD and pro-IL-1β and pro-IL-18, forming an unselective pore on the cytomembrane, secreting IL-1β and IL-18 and influx of water, causing cell death. Non-canonical pathway initialed by caspase-4/5/11 which is activated by LPS. Active caspase-4/5/11 activates caspase-1 and cleaves GSDMD directly, leading to pyrotosis

The fact that macrophage polarization does have a great effect on PF is generally proven [6]. Similarly, macrophage pyroptosis, because of the release of pro-inflammatory cytokines and regulating polarization, is considered to relate to PF (Fig. 2). CCL2, the classical inflammatory cell chemokines, not only plays an important role in boosting inflammation in the early period of PF but has a profibrotic capacity [63]. Growth evidence proved the notion that CCL2 can rise the expression of ECM and promote the TGF-β released by fibroblasts in the pulmonary, stimulating the deposition of collagen [63]. Additionally, CCL2, by activating the ERK1/2 pathway, contributes to the release of IL-6 which efficaciously restrains the death of fibroblasts via the IL-6/STAT3 pathway [30, 64]. The conception that the NLRP3-inflammasome which participates in macrophage pyroptosis is associated with PF is widely accepted [16, 65–67]. On the one hand, NLRP3-inflammasome, as the most classical inflammasome of pyroptosis, induce the inflammatory death of macrophages. On the other hand, it has been demonstrated that this inflammasome promoted the epithelial interstitial transformation in bleomycin-induced fibrosis by regulating levels of TGF-β [68–72]. NOD-like receptors are widely expressed in the cytoplasm of immune cells such as monocytes, lymphocytes and NK cells and have the function of identifying damage associated molecular patterns (DAMPs) and pathogen associated molecular patterns (PAMPs) and participating in the pyroptosis and the polarization of macrophages [73, 74]. Research in 2022 reported that the expression level of M1 macrophage and the NLRP-related protein casp-1 are decreased by applying discoidin domain receptor-1 inhibitor (DDR1-I) in Raw264.7 macrophages in vitro [75]. Additionally, researchers found that oleamide shows the capacity of activation of NLRP3-inflammasome, consequently promoting polarization of M0 into M1 [76]. And the activation of NLRP3-inflammasome is one of the most important procedures of pyroptosis [44]. Also, the TLR4/NK-κB pathway working as a classical inflammatory pathway is not only associated with macrophage polarization but participating in macrophage pyroptosis [57]. TLR4 which is classically activated by DAMPs recruits downstream myeloid differentiation factor (88MyD88) by homophile interaction [77]. MyD88 is a type of cytosolic-soluble protein, which has the ability to phosphorylate IKKβ and further phosphorylate I-κB leading to the forfeit of bioactivity of suppression κB [78]. The final effect of this course is the release of the P65 / P50 NF- κ B dimer and the promotion of the transcription of NLRP3-inflammasome as well as other inflammatory factor precursors [79]. After completion of transcription of NLRP3-inflammasome, it will mediate the pyroptosis of macrophages [80]. Pyroptotic macrophages will release inflammatory cytokines IL-1β which is an important regulatory factor in polarization [53, 74]. With pretreated with low concentrate of Pirfenidone for 24 h, researchers found that the polarization of M2 was inhibited in Raw264.7 cells because of downregulation of NF-κB p50 [81].

A conclusion that can be drawn from those researches is that there is a potential association between macrophage polarization and pyroptosis, and both of their effect on fibrosis of pulmonary. A deeper mechanistic understanding of this relationship which needs to be further researched would offer an innovative and more precise targeted therapy strategy for PF.

### The macrophage of other death forms in PF

#### Macrophage apoptosis in PF

**Fig. 3:**
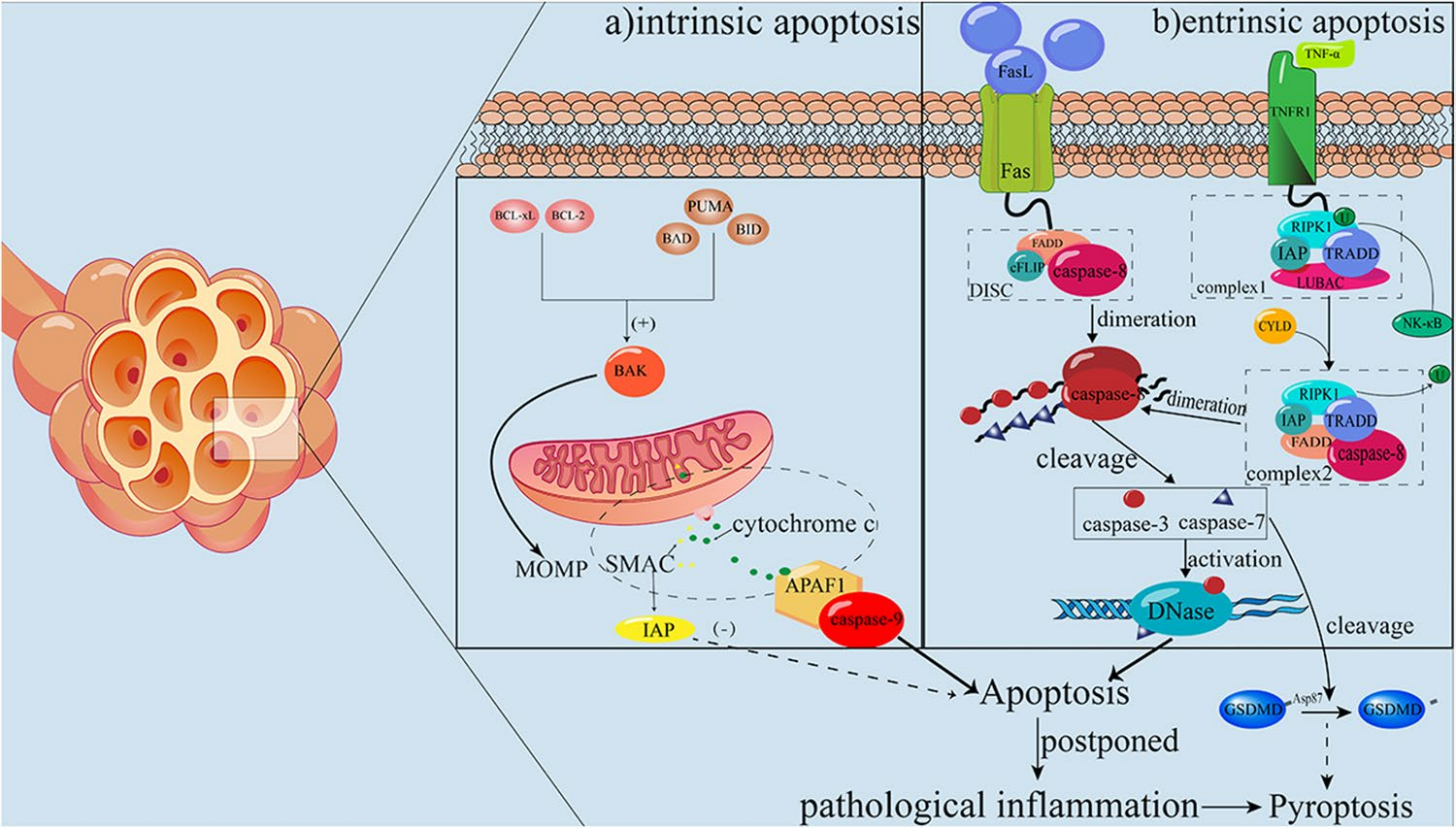
Apoptosis is mediated by intrinsic and extrinsic pathways. **a** Intrinsic pathway; **b** Extrinsic pathway

Macrophage apoptosis is associated with PF (Fig. 3). Compared with pyroptosis, apoptosis, which is mediated by two certain pathways: the intrinsic and the extrinsic pathway, is traditionally considered as a non-immunologically linked form of cell death [19]. Research in 2016 reported that Grp78 as the major unfolded protein response regulator shows the ability to suppress macrophage apoptosis, consequently exacerbating bleomycin-induced PF [82]. Moreover, after being disposed of by LPS, researchers found that PF in a mouse model of silicosis was aggravated because of promoted apoptosis and inflammation in AMs [83]. Of note, apoptosis-relating caspase-3/-7 can hinder pyroptosis in the way of cleaving the non-inflammatory site Asp87 of GSDMD [46]. Postponed apoptosis of macrophages conversely induces pathological inflammation and continuously boosts pro-inflammatory cytokines release [45, 84]. TREM-1 knockout mice show diminishing inflammation in the LPS-induced pulmonary injury model [85]. Mice are protected from bleomycin-induced PF once c-FLIP, an anti-apoptotic protein, is deleted from CD11bhi macrophages [86]. In conclusion, excessive infiltration of macrophages promotes PF and suitable repression of this effect would be a novel research direction in treating PF in the future.

#### Macrophage autophagy in PF

**Fig. 4:**
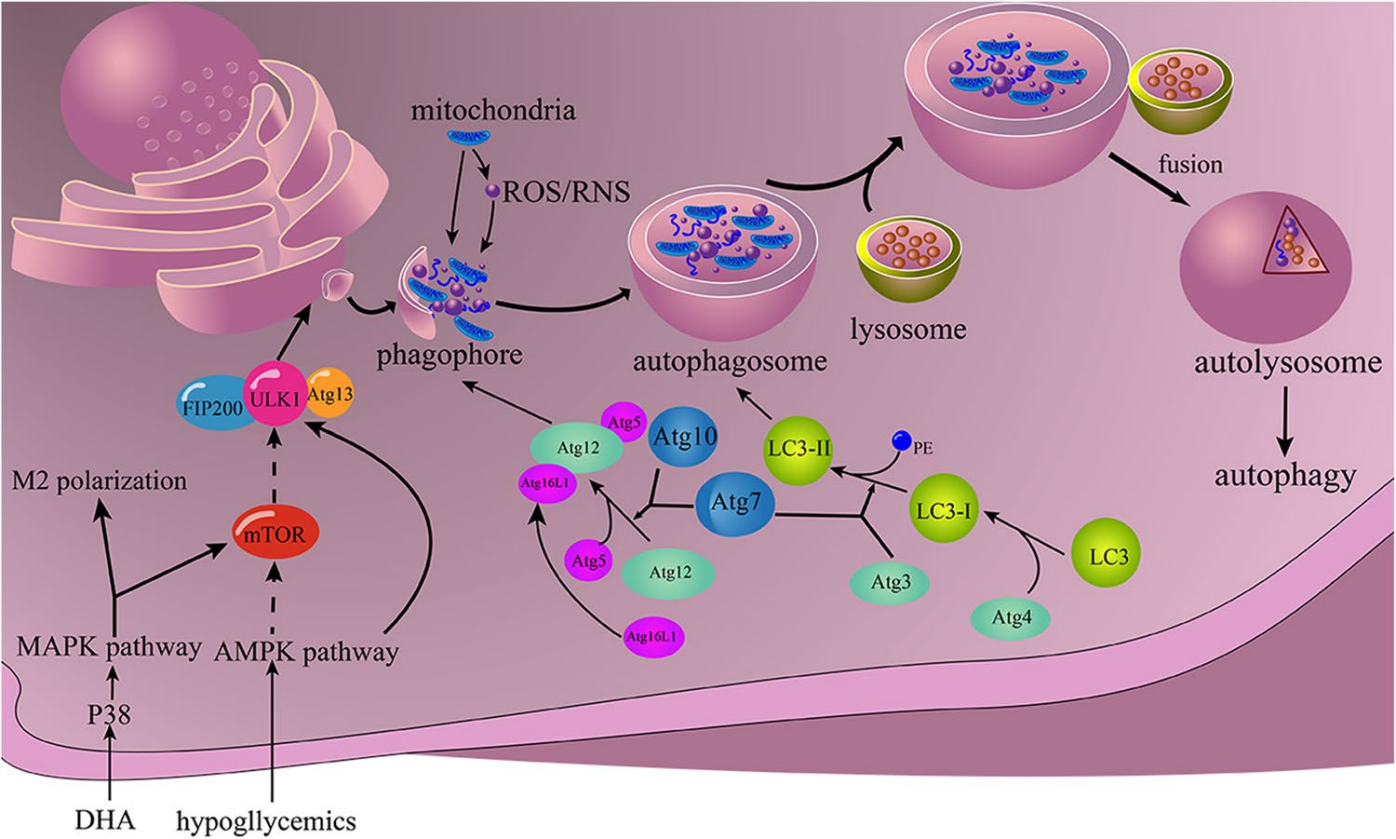
The main process of autophagy is the formation of autolysosomes

Autophagy, which is mediated by the mechanistic target of Rapamycin (mTOR), the ER stress, the insulin pathway, and a variety of other pathways, features as a form of catabolic cellular components that is highly conserved and autophagosome-dependent (Fig. 4.) [87, 88]. In addition, to contribute in sustain the metabolic balance of cells, autophagy has also been shown a relationship with macrophage pyroptosis in PF [89–91]. Recent research reported that Resolvin D2 (RvD2), an anti-innate-immune mediator, promoted the degradation of NLRP3-inflammasome with the indeterminate mechanism, however [92]. Moreover, the loss of autophagy-related proteins was observed in the capacity of augmenting the release of IL-1β and pyroptosis in 2008 [93]. PAMPs and DAMPs activated autophagy by PRRs signaling pathway downregulates pyroptosis via eliminating the cleaved GSDMD produced by caspase which may be related to the AMPK-eEF-2 K pathway [18]. DAMPs, like HMGB1 and interleukin, initial and promote local inflammation in the early stage of fibrosis and ROS, reactive nitrogen species (RNS), and mitochondrial DNA (mtDNA) have the ability to induce autophagy by causing mitochondria damage [94, 95]. Damaged or dysfunctional mitochondria can release ROS and mtDNA, forming a cascade of pro-pyroptotic factors, resulting in NLRP3-inflammasome excessive activation. However, those factors initial mitophagy meanwhile, activating the mitochondrial autophagosome, following decomposing the damaged mitochondrial, leading inhibition of pyroptosis. RNS, similar to ROS, is responsible for the promotion of pyroptosis, whose clearance may abate macrophage pyroptosis via autophagy [94, 96–98]. After the knockout of the autophagy-related protein-7 (Atg7) gene, the activity of the inflammasome of macrophage and the level of serum IL-1β are raised in mice models of pseudomonas aeruginosa-induced sepsis [99]. All these findings indicate that autophagy has an excellent ability to eliminate inflammation. which may link with suppressing macrophage pyroptotic death.

Autophagy mediates PF via multi-pathway on the other hand. In the acute inflammatory phase of PF, autophagy is widely activated, to clear the invading pathogenic substances [100, 101]. However, it had been reported that, in the lung tissue of patients with PF, the autophagosomes were remarkably decreased [102, 103] and ubiquitinated proteins were accumulated in cells [12], which indicated that autophagy is inhibited in the fibrotic phase of this disease which leads to disability of removing ECM and returning fibrosis of pathological pulmonary. Chemokine (C-X3-C motif) receptor 1(CX3CR1), expressed on the macrophage, NK cells and T lymphocytes, is the receptor for CX3CL1 and is important to induce the generation of ROS and macrophage autophagy [104]. The overexpression of CX3CL1 promoted fibrosis in a mouse model of hyperoxic lung injury, which is related to the activation of Akt1-mediated autophagy of macrophage. By further using CX3CL1 inhibitor, 3-methyladenine (3-MA), macrophage autophagy, and fibrosis of pulmonary were reduced [105]. The classical hypoglycemics metformin is recently found to be able to activate the AMPK-mTOR pathway, thereby attenuating PF in silicosis [106]. Besides, autophagy exacerbates fibrosis via promoting M2 macrophage polarization. DHA (docosahexaenoic acid) enhances the transformation of M2 through autophagy and the p38-MAPK pathway [107]. Ubiquitin-specific protease 19 (USP19), as a type of deubiquitinating enzyme, positively effect M2-like macrophage polarization by the autophagy-related response to NLRP3 [108]. However, interestingly, researchers found that isoprenaline can down-regulate autophagy by activating ROS-ERK and mTOR signaling pathways, enhancing M2 macrophage polarization [109].

In summary, a growing number of researches demonstrate the robust relationship between autophagy and pyroptosis in PF. It is clear that autophagy participates in process of PF in a pyroptosis-dependent or M2 macrophage-dependent way. Nevertheless, the question of whether are there more interaction between autophagy and fibrosis is open yet.

### The crosstalk between the signaling pathway of pyroptosis, apoptosis and autophagy

**Fig. 5:**
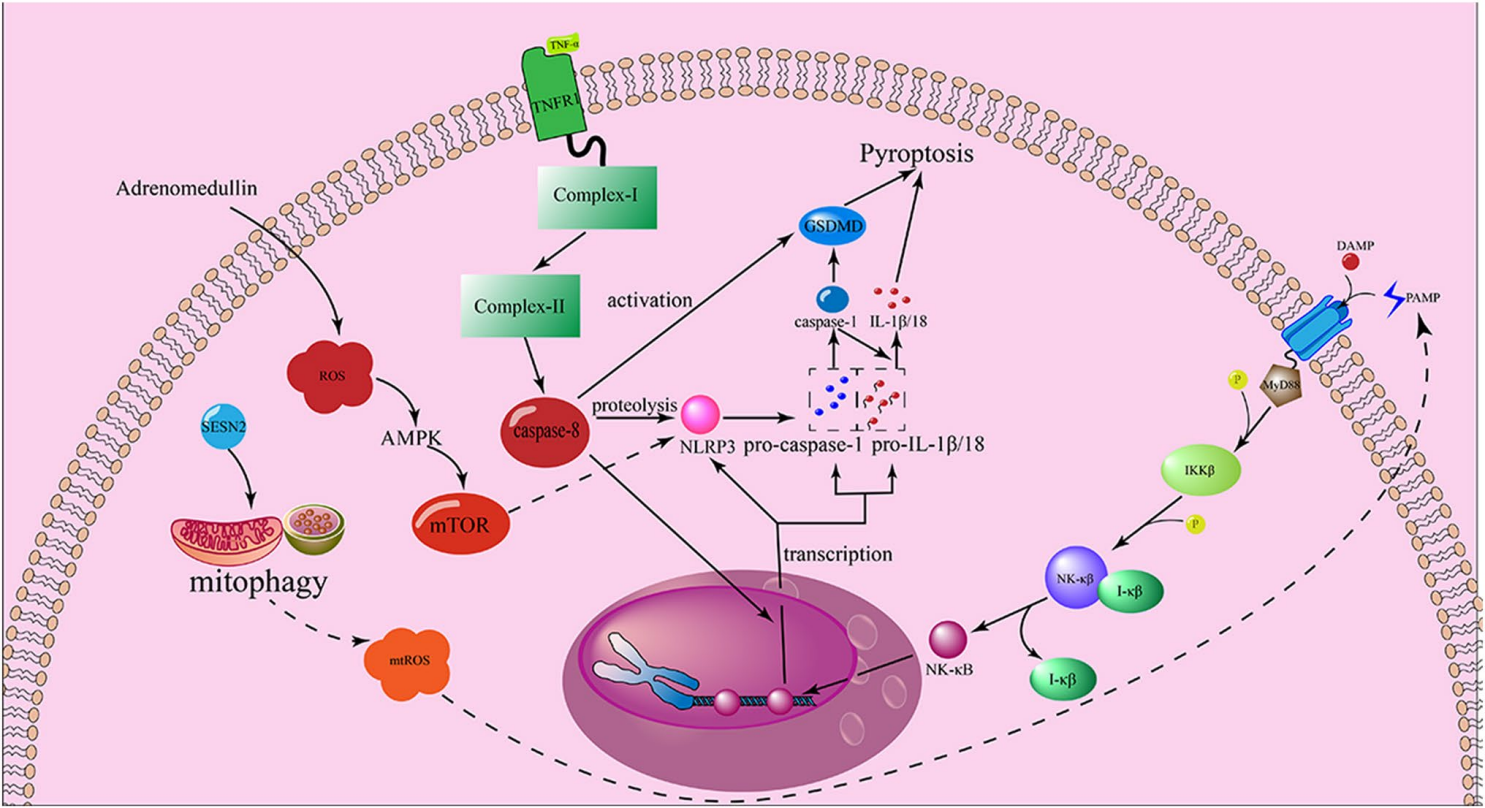
The crosstalk between the signaling pathway of pyroptosis, apoptosis, and autophagy. DAMPs and PAMPs can activate the corresponding receptor, recruiting cytosolic MyD88, and starting the NK-κB pathway. In the process of post-transcription of NLRP3, pro-caspase-1 and pro-IL-1β/18, the apoptotic caspase-8 can promote the activation of GSDMD and induce the proteolysis of NLRP3 and contribute to the transcription of NLRP3, pro-caspase-1 and pro-IL-1β/18. SESN2 can induce mitophagy which releases the mtROS which acts as a PAMP. Adrenomedullin promotes autophagy via ROS/AMPL/mTOR pathway, abating pyroptosis of the cell

Growth number of researches have been established a more complex mode of cell death and as crosstalk among them [18, 46]. To make a deep understanding of those forms of cell death in PF, it is necessary to elucidate some essential signal pathways and the crosstalk of them (Fig. 5). The term inflammasome was first described in 2002 [110]. It had been widely proven in past decades that the nod-like receptor family and pyrin and HIN domain family were involved in the formation of an inflammasome [111–113]. The NLRP3 is greatly important for human immune defenses, whose activation may relate to multifactor. The NLRP3 is mainly composed of 3 domains: C-terminal leucine-rich repeats (LRR), a nucleotide-binding oligomerization domain (NACHT) domain and the N-terminal pyrin domain (PYD). The NLRP3 recruits and activates caspase-1 by binding ASC [114]. The activation of NLRP3 which includes initial up-regulation of NLRP3 and the level of pro-IL-1β is essential for this procedure [115]. To date, it was thought that k+ outflowing, ROS and lysosomal damage are the three major hypotheses in the field of activation of NLRP3, however, the precise mechanism is in controversial yet [116–118]. In fact, some of researches suggest that NK-κB plays a specific role in the activation of NLRP3 [119, 120]. Once activated by DAMPs or PAMPs, TLRs recruit downstream MyD88 which phosphorylates IKKβ via the IRAK-TRAF6-NIK pathway and then further phosphorylates I-κB, leading to the deprivation of bio-activity of inhibiting κB, which final causing the transcription of NLRP3-inflammasome and the precursor of other inflammatory cytokines [120, 121]. The inflammasomes need to undergo several other post-translational modifications including ubiquitination [122] and sumoylation [123] before it is activated. After the assembly of NLRP3-inflammasome is finished, caspase-1 would be activated in certain vitro conditions [71, 124]. However, new researches also indicated that TLR4/MyD88 signaling pathway can initial NLRP3-inflammasome through a non-transcriptional mechanism which may relate to the production of mtROS [125].

Distinct from pyroptosis, caspases that participate in apoptosis can be functionally classified as initiator caspases, including caspase-8 and -9 and executioner caspases, including caspase-3 and -7 [126–128]. Tumor necrosis factor receptor 1 (TNFR1) activated by TNF-α recruits the RIPK1 which is ubiquitinated after the complex-I is formed, and RIPK1 will be deubiquitinated if the inhibitor of apoptosis proteins are lacking and deubiquitinated RIPK1 forms complex-II, then activating pro-apoptotic caspase-8, one component of complex-II, finally causing apoptosis [19]. Caspase-8 is the major part of the connection between pyroptosis and apoptosis. Caspase-8 not only has the ability of proteolysis to process caspase-1 but also plays an integral role in the transcription of NLRP3 and IL-1β [129]. Furthermore, caspase-8 can directly activate GSDMD whose cleavage participates in the formation of NLRP3 and production of IL-1β during the infection of Yersinia, following the blockade of TAK1 [130]. Caspase-1 also is the evidence for the relativity between pyroptosis and apoptosis, in 2008 when it was observed that caspase-1 can cleave caspase-7 in macrophages [131]. Researchers found that caspase-1 can activate caspase-3 in the condition of deficiency of GSDMD [132].

Similar to apoptosis, it has been found that signaling pathways regulating autophagy are involved in regulating pyroptosis [18]. The mTOR pathway plays a more crucial role in the autophagy of cells. Insulin and IGF activate the RTKs and activate the PI3K-AKT signaling pathway, the activated AKT phosphorylates TSC1/2 which dissociates from lysosome and subsequently activates Rheb, the activator of mTORC1 [133, 134]. Interestingly, AKT also participates in the modulation of the mTORC2 pathway of which mechanism is still not completely clear [135]. The overproduction of ROS can not only activate NLRP3 inflammasome which has been mentioned above but induces autophagy also [136]. Researchers found that the pyroptosis of Leydig cells was alleviated after being processed by adrenomedullin by promoting autophagy via the ROS/AMPK/mTOR pathway [137]. SESN2, a stress-derived protein, inhibits activation of NLRP3-inflammasome in macrophage, by inducing mitophagy which is a form of autophagy [138]. Cytosolic DNA acts as the activator of stimulator of interferon genes (STING) via the GMP/cGAMP pathway which is the classical pathway of inducing autophagy [139].

Collectively, the crosstalk between apoptosis, autophagy and pyroptosis has been further studied in recent years but some of the mechanisms still are unclear. More researches are needed to conduct on the crosstalk between pyroptosis and the other two cell death forms, especially in the field of PF. In the past, we often take the impact of only one mode of cell death into consideration in PF, while simultaneously neglecting the potential effect of other forms of cell death. An all-inclusive understanding of cell death happening in the process of PF will provide a novel direction of research in PF which possibly contributes to the treatment and quality of life of patients with PF.

## Conclusion

Lung M1 and M2 macrophages are distinct cell subtypes and are both involved in the pathogenesis of PF. M1 and M2 macrophages play different roles in the pathogenesis of PF. Generally, M1 macrophages are responsible for wound healing after alveolar epithelial injury, while M2 macrophages are determined to over repair the damaged tissue and terminate the degradation of ECM in the lung [9]. A variety of regulatory cytokines, chemokines, mediators and immune-regulatory cells affect macrophage polarization in the lung [9]. Studies have provided evidence for a connection between cell death and macrophage polarization and understanding of the impact of macrophage death on ECM accumulation is critical in fully elucidating the mechanisms underlying PF. Following an initial event of chronic persistent injury, cell death and inflammation can induce each other and drive a release of regulatory cytokines, chemokines, mediators that lead to exaggerated fibrosis effects [10, 102]. Macrophage pyroptosis can activate the release of signaling pathways caspase-1 and IL-1 and promote the secretion of TGF-β1 [43], thus promoting the proliferation and differentiation of myofibroblasts and inflammatory response. Apoptosis and autophagy were thought to be the form of cell death during homeostasis and development and has been heavily studied and discussed in numerous pieces of literature on PF [19, 101, 102]. The gaps in our knowledge of cell death include whether different types of cell death signaling developed separately as responses to specific triggers or whether they represent parts of a signaling network that follow common regulatory mechanisms.

Although therapies for PF included a variety of drugs and non-pharmacological interventions remain unsolved regarding the exact mechanisms of manipulating the balance of M1/M2 phenotype in PF pathogenesis and are unable to effectively attenuate PF [3]. Comprehensive understanding of the molecular mechanisms that regulate cell death will allow the development of strategies that control cell death, thereby developing novel interventions for PF.

## Data Availability

Not applicable.

## Abbreviations

PF: Pulmonary fibrosis
ECM: Extracellular matrix
AECs: Alveolar epithelial cell
TNF-α: Tumor necrosis factor-α
NF-κb: Nuclear factor-kappa B
ScRNA-seq: Single-cell RNA-sequencing
PDGF: Single-cell RNA-sequencing
VEGF: Vascular endothelial growth factor
IGF-1: Insulin like growth factor
FGF: Fibroblast growth factor
CCL: Chemokine (C-C motif) ligand
MMP: Matrix metalloproteinases
IPF: Idiopathic PF
GM-CSF: Granulocyte-macrophage colony stimulating factor
ROS: Reactive oxygen species
LPS: Lipopolysaccharide
ACS: Apoptosis-associated speck-like protein
NLRs: Nod-like receptor
PRR: Pattern recognition receptor
CARD: Caspase recruitment domain protein
GSDM: Gasdermin
TLR: Toll-like receptor
HMGB1: High mobility group box 1
RAGE: Receptor for advanced glycation end products
STAT: Signal transducer and activator of transcription
DAMP: Damage associated molecular pattern
PAMP: Pathogen associated molecular pattern
MyD88: Myeloid differentiation factor 88
IKKB: I-kappa-B kinase B
BCL-xL: B-cell lymphoma extra large
BCL-2: B-cell lymphoma 2
BAK: BCL-2 antagonist/killer 1
MOMP: Mitochondrial outer membrane permeabilization
SMAC: Second mitochondria–derived activator of caspase
APAF1: Apoptotic protease activating factor 1
Asp: Aspartic acid
Fas: CD95
FasL: Fas ligand
FADD: Fas-associated death domain
cFLP: Cellular FLICE-inhibitory protein
IAP: Inhibitor of apoptosis protein
DISC: Death-inducing signaling complex
DNase: Deoxyribonuclease
RIPK1: Receptor-interacting protein kinase 1
TRADD: TNFR1-associated death domain
mTOR: Mechanistic target of rapamycin
RVD2: Resolvin D2
Atg: Autophagy-related protein
AMPK: AMP-activated protein kinase
MAPK: Mitogen-activated protein kinase
RNS: Reactive nitrogen species
mtDNA: Mitochondrial DNA
CX3CR1: Chemokine (C-X3-C motif) receptor 1
3-MA: 3-Methyladenine
DHA: Docosahexaenoic acid
USP19: Ubiquitin-specific protease 19
TNFR1: Tumor necrosis factor receptor 1
STING: Stimulator of interferon genes
LRR: Leucine-rich repeats
NACHT: Nucleotide-binding oligomerization domain
